# A systematic review on the role of microbiota in the pathogenesis and treatment of eating disorders

**DOI:** 10.1192/j.eurpsy.2020.109

**Published:** 2020-12-16

**Authors:** Elvira Anna Carbone, Pasquale D'Amato, Giuseppe Vicchio, Pasquale De Fazio, Cristina Segura-Garcia

**Affiliations:** 1 Department of Health Sciences, University “Magna Graecia”, Catanzaro 88100, Italy; 2 Outpatient Service for Clinical Research and Treatment of Eating Disorders, University Hospital Mater Domini, Catanzaro 88100, Italy; 3 Department of Pharmacy, Health and Nutritional Sciences, University of Calabria, Rende 87036, Italy; 4 Department of Medical and Surgical Sciences, University “Magna Graecia”, Catanzaro 88100, Italy

**Keywords:** Anorexia nervosa, binge eating disorder, bulimia nervosa, eating disorders, microbiota

## Abstract

**Background:**

There is growing interest in new factors contributing to the genesis of eating disorders (EDs). Research recently focused on the study of microbiota. Dysbiosis, associated with a specific genetic susceptibility, may contribute to the development of anorexia nervosa (AN), bulimia nervosa, or binge eating disorder, and several putative mechanisms have already been identified. Diet seems to have an impact not only on modification of the gut microbiota, facilitating dysbiosis, but also on its recovery in patients with EDs.

**Methods:**

This systematic review based on the PICO strategy searching into PubMed, EMBASE, PsychINFO, and Cochrane Library examined the literature on the role of altered microbiota in the pathogenesis and treatment of EDs.

**Results:**

Sixteen studies were included, mostly regarding AN. Alpha diversity and short-chain fatty acid (SCFA) levels were lower in patients with AN, and affective symptoms and ED psychopathology seem related to changes in gut microbiota. Microbiota-derived proteins stimulated the autoimmune system, altering neuroendocrine control of mood and satiety in EDs. Microbial richness increased in AN after weight regain on fecal microbiota transplantation.

**Conclusions:**

Microbiota homeostasis seems essential for a healthy communication network between gut and brain. Dysbiosis may promote intestinal inflammation, alter gut permeability, and trigger immune reactions in the hunger/satiety regulation center contributing to the pathophysiological development of EDs. A restored microbial balance may be a possible treatment target for EDs. A better and more in-depth characterization of gut microbiota and gut–brain crosstalk is required. Future studies may deepen the therapeutic and preventive role of microbiota in EDs.

## Introduction

There is growing interest in factors contributing to the genesis of eating disorders (EDs), supported by the great impact on patients’ quality file and burden [1]. The etiology of EDs is multifactorial due to the presence of predisposing, precipitating, and perpetuating factors that allow the onset and maintenance of the disorders [2].

Recently, research has focused on the microbiota [3] and its composition, with 100 trillion microbial cells residing in different human body areas [4]. The two phyla “Bacteroidetes” and “Firmicutes” represent about 90% of the bacterial populations identified, whereas the remaining 10% is mainly composed of Actinobacteria and Proteobacteria [5,6]. The phylum Firmicutes is represented by more than 200 different genera: *Lactobacillus*, *Bacillus*, *Enterococcus*, *Ruminococcus*, and *Clostridium.* Bacteroidetes essentially consists of two predominant genera, the *Bacteroides* and the *Prevotella.* *Bifidobacterium* belongs to the Actinobacteria. A preponderance of *Lactobacilli* has been detected in the area of the stomach and duodenum and *Streptococci* at the jejunal level, whereas the ileocolic regions show a profound heterogeneity of bacterial species, including *Lactobacilli*, *Escherichia coli*, and other Enterobacteria, *Enterococci faecalis*, Bacteroides, *Bifidobacteria*, *Peptococci*, *Petostreptococci*, *Ruminococci*, and *Clostridia* [5]. The composition of microbiota is not stable during life: presents rapid changes from early childhood, stabilizes in adulthood, and then deteriorates in old age [7,8]. Different factors contribute to both lifetime variation and stability of the gut microbiota (i.e., age, sex, ethnicity, geographical location, environment, climate, delivery mode, breastfeeding, weaning, body mass index (BMI), exercise, smoking, alcohol, drugs, and diet) [9,10].

Evidence highlighted that the alteration in the normal microbial composition, called dysbiosis, may contribute to the development of EDs when associated with a specific genetic susceptibility [11–16], and several putative mechanisms have already been identified. Furthermore, nutritional rehabilitation represents one of the essential focuses for EDs, and the intake of macronutrients can significantly affect the composition of microbiota [17,18], reducing dysbiosis. To date, therapeutic strategies that can correct the microbiota are represented by fecal microbiota transplantation (FMT) [19], but the use of prebiotics and probiotics to restore microbiota alterations has also been proposed [20,21].

A recent research and systematic review demonstrated that gut dysbiosis may represent hallmarks in AN [22] suggesting the potentially interesting therapeutic targets.

Nevertheless, there are no review focusing on the other ED as bulimia nervosa (BN) or binge eating. Thus, in order to fill this gap, we aimed to update and critically analyze the existing literature on the possible role of altered microbiota in the etiopathogenesis and treatment of patients with EDs.

## Methods

This systematic review was done according to Participants Intervention Comparator and Outcome (PICO) methodology, and quality was measured by means of Grading of Recommendations Assessment, Development and Evaluation (GRADE) [23].***Structured question:*** Does dysbiosis play a role in the pathophysiological development and outcome of EDs?

### Inclusion criteria

- ***P**articipants.* The review considered studies that included participants diagnosed with anorexia nervosa (AN), BN, binge eating disorder (BED), or ED not otherwise specified.
- ***I**ntervention(s).* This review considered studies that evaluate qualitative and quantitate microbiota analysis in EDs with/without a pathogenesis implication and studies that evaluate microbiota dysbiosis in EDs with/without the use of probiotics/prebiotics/microbiota transplantation.
- ***C**omparator(s).* This review considered studies that compare the intervention in outpatients and inpatients to other ED or health control (HC) group.
- ***O**utcomes.* This review considered studies that evaluated if dysbiosis accounts for eating symptoms, maintenance, or treatment of the disorders. Various instruments are likely to be used to measure these outcomes. This review focused on those using validated questionnaires/tools as patient-reported outcome measures, measures of mood, anxiety, and eating psychopathological symptoms.
- *Types of **s**tudies.* To present a complete overview of the literature, we included randomized and nonrandomized, qualitative, and quantitative studies with and without comparison groups, case reports, and observational studies with any sample size.

### Exclusion criteria

Studies were excluded in the following cases: studies on animals; patients with EDs due to other medical conditions or induced by substances; pregnant or postpartum women; patients with digestive disease (i.e., inflammatory bowel disease, irritable bowel syndrome, and coeliac disease); patients undergoing other psychiatric and/or metabolic treatments that could modify affectivity, weight, and appetite; or patients receiving nonstandard medications or any other therapy (i.e., antibiotics or steroids). Handbooks, manuals, editorials, letters to editor, reviews, or meta-analyses were also excluded. If duplicated data were found, datasets with the highest number of participants were included. Only eligible publications meeting the inclusion criteria have been included and cited in this review.

### Search strategy

Articles published up to August 1, 2020, were retrieved from PubMed, EMBASE, PsychINFO, and the Cochrane Library, on human data without language or time restriction, based on the PICO strategy.

Gray literature and unpublished studies were also considered. The following MESH search strings were used: (Microbiota OR dysbiosis OR gut microbiota OR intestinal microbiota OR gastrointestinal microbiota OR microbial metabolites OR microbial peptides OR microflora OR probiotics OR prebiotics OR FMT) AND (EDs OR feeding OR anorexia OR AN OR bulimia OR BN OR binge eating OR BED) AND (etiopathogenesis OR cause OR etiology OR pathogenesis OR pathophysiological).

**Figure 1.**
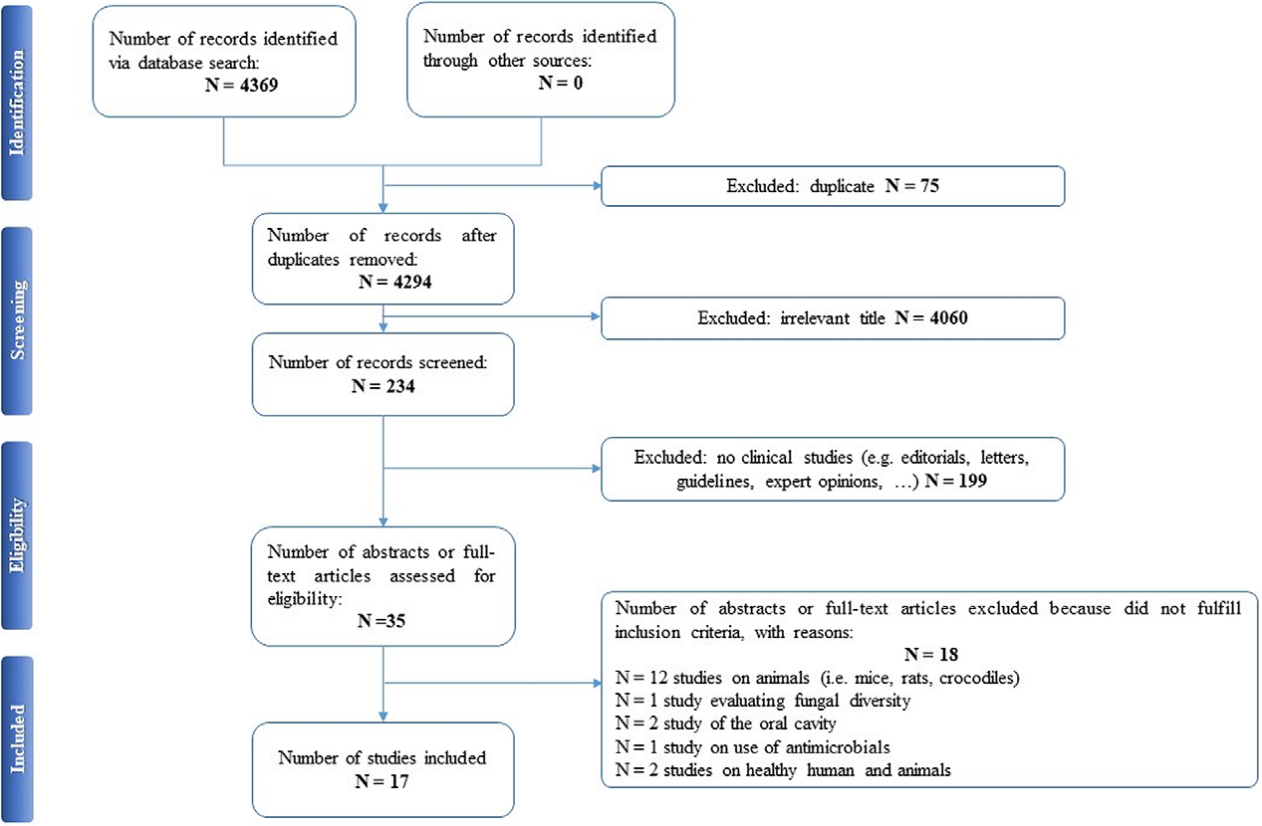
The PRISMA flow chart.

Selected articles were reviewed independently by two authors (EAC and PDA), who screened the titles and abstracts and read the full texts of any articles that met the eligibility criteria. In case of any disagreement, consensus was reached through discussion. All relevant original publications obtained from the literature search were identified, and the full texts were retrieved and reviewed. The reference lists were screened, and additional data were included. Preferred Reporting Items for Systematic Reviews and Meta-Analyses (PRISMA) criteria and recommendations were followed to improve the clarity and plainness of the review process [24]. Figure 1 shows the research strategy.

### Assessment of quality

The quality of the studies was assessed using the GRADE approach [23]. Two reviewers (EAC and CSG) used GRADE approach to assess the quality of evidence from five items of research limitations, inconsistency, indirectness, inaccuracy, and publication bias.

The quality of evidence was rated as “high,” “moderate,” “low,” or “very low” based on GRADE rating standards. “High” quality of the evidence indicates that future research is very unlikely to change existing evidence, “moderate” indicates that future research may change the results, “low” level indicates that future research is likely to change the evaluation results, having an important impact on existing evidence, whereas “very low” indicates highly uncertainty about the existing evidence. In this review, GRADE ratings ranged from moderate to low or very low quality of evidence. The quality assessment was finally reviewed and agreed by the whole review team.

## Results

Our search strategy resulted in 4,369 papers. After removing duplicates and irrelevant titles, only 234 articles were considered sufficiently relevant to warrant abstract review. Among them, 199 were excluded, because they were editorials, letters, reviews, systematic reviews, meta-analyses, guidelines, expert opinions, or different interventions, leaving 35 to be assessed for eligibility, with relevant references within these publications also identified and reviewed. Following the screening of literature according to inclusion and exclusion criteria, 18 studies were excluded, because they did not fulfill the inclusion criteria; the remaining 17 were included in the review. Table 1 describes the characteristics of these 17 studies.

**Table 1.** Main characteristics of included studies.

Author, year	Study design	Criteria	Sample	Target phyla/genera/families/other	Measures	Psychiatric assessment	Main results	Comment
Armougom, 2009 [25]	Cross-sectional observational Grade: **	DSM-IV	*N* = 49	Firmicutes (*Lactobacillus*), Bacteroidetes, and *Methanobrevibacter smithii.*	Fecal sample	N/A	Low Bacteroidetes community among obese patients. Higher *Lactobacillus* species concentration in obese patients > HC > AN. Much higher *M. smithii* in AN > HC.	First study on the human gut microbiota for the identification of specific patient microbial communities and the confirmation of a specific profile.
			20 obese		Real-time PCR assay			
			9 AN					
			20 normal weight HC					
Million, 2013 [26]	Cross-sectional observational Grade: **	DSM-IV	*N* = 263	Bacteroidetes, *M. smithii*, Firmicutes as *Lactobacillus* species (*Lactobacillus reuteri*), *Escherichia coli*, and *Bifidobacterium animalis.*	Fecal sample	N/A	*L. reuteri* positively correlated with BMI. *B. animalis*, *M. smithii*, and *E. coli* negatively associated with BMI.	It confirms the link between diet, changes in the microbiota, and correlation with BMI or weight gain.
			134 obese		Real-time PCR assay			
			38 overweight					
			76 lean 15 AN					
Pfleiderer, 2013 [27]	Case report Grade: *	Not specified	A 21-year-old Caucasian AN-R female	133 species of bacteria: 79 Firmicutes species, 25 Actinobacteria species, 18 Bacteroidetes species, and 11 Proteobacteria species.	Fecal sample	N/A	Identification of 133 bacterial species, with 19 bacterial species never isolated from the human gut before, including 11 new bacterial species that belonged to Ruminococcaceae, Lachnospiraceae, and Erysipelotrichaceae.	It revealed new bacterial species participating significantly to the extension of the gut microbiota repertoire.
					Culture growth and mass spectrometry (MALDI–TOF)			
Gouba, 2014 [28]	Case report Grade: *	Not specified	A 21-year-old Caucasian severe AN female	28 eukaryotic species: 17 Viridiplantae sp., 8 fungi (*S. cerevisiae, P. solitum, C. bruhnei, C. capitatum, Sclerotium sp., M. pachydermatis, M. restricta*, and *M. globosa*), 2 metazoan (*M. trossulus* and *M. galloprovincialis*), and 1 protozoan (*Tetratrichomonas spp.*).	Fecal sample	N/A	Restricted diversity of fungi. 4 new microeukaryotes previously undescribed in the human gut: *Tetratrichomonas sp.*, *Aspergillus ruber*, *Penicillium solitum*, and *Cladosporium bruhnei.*	Lower fungi diversity in AN. Establishing microeukariote repertoire in gut microbiota is necessary to better understand its role in human health.
					Culture and PCR			
Kleiman, 2015 [29]	Longitudinal Grade: ***	DSM-IV	*N* = 28 AN	Verrucomicrobia, Saccharibacteria, Tenericutes, Proteobacteria, Fusobacteria, Firmicutes, Cyanobacteria, Bacteroidetes, and Actinobacteria.	Fecal sample	BDI-II	AN: Significant changes t_1_–t_2_ in taxa abundance and beta (between-sample) diversity. AN significantly lower alpha (within-sample) diversity than HC (both t_1_ and t_2)_. Differences in taxa abundance between AN and HC. Depression, anxiety, and eating disorder psychopathology at t_1_ significantly associated with composition and diversity of gut microbiota.	It demonstrated intestinal dysbiosis in AN and an association between mood and the gut microbiota in AN.
			16 (t_1_)–10 (t_2_)		Anthropometric assessments	BAI		
						EDI-2		
					Ultrasound measurement of SAT thickness			
						EDE-Q		
			12 HC					
Morita, 2015 [14]	Cross-sectional observational Grade: **	DSM-IV	*N* = 46	*C. coccoides, C. leptum, B. fragilis*, *Bifidobacterium*, *Atopobium*, *Prevotella*, Enterobacteriaceae, *Enterococcus*, *Staphylococcus*, *Streptococcus*, *C. difficile, C. perfringens, Lactobacillus spp.* (*L. gasseri, L. reuteri, L. ruminis, L. plantarum, L. sakei, L. casei, L. brevis*, and *L. fermentum*).	Fecal sample through the Yakult Intestinal Flora-SCAN	N/A	AN: significantly lower total bacteria, obligate anaerobes (*Clostridium coccoides*, *Clostridium leptum*, and *Bacteroides fragilis*), *Streptococcus*, and *Lactobacillus plantarum.* Lower acetic and propionic acid concentrations in the feces especially in AN-R (AN-R < AN-P < HC). AN-R: lower *Bacteroides* fragilis and *Clostridium coccoides.*	It showed gut dysbiosis in AN with clear difference in the bacterial components between the AN patients (considering subgroups) and HCs.
			25 AN					
			(14 AN-R and 11 AN-P)					
			21 HC					
					SCFAs and pH of feces (chromatography)			
					Blood sample			
Breton, 2016 [30]	Cross-sectional observational Grade: **	Not specified	N = 95	Not specified.	Fecal sample	EDI-2	ClpB was readably detectable in plasma of all participants. ClpB elevated in ED patients, without differences according to diagnosis. ClpB correlated with EDI-2 and MADRS.	ClpB plasma concentrations can be elevated in EDs patients and associated with ED-related psychopathological traits supporting a link between bacterial ClpB and the ED pathophysiology.
			24 AN		Blood sample: ClpB protein plasma concentration, α-MSH, of anti-ClpB IgG, IgM, and α-MSH-reactive IgG plasma levels	MADRS		
			29 BN					
			13 BED					
			29 HC					
Mack, 2016 [31]	Longitudinal Grade: ***	Not specified	*N* = 110 AN	Bacteroidetes, Firmicutes, Actinobacteria, Proteobacteria, and Verrucomicrobia.	Fecal sample	N/A	AN: lower Bacteroidetes at t_1_ and at t_2_, while Firmicutes increased from t_1_ to t_2_. AN: higher mucin-degraders and members of *Clostridium* clusters and reduced levels of the butyrate-producing *Roseburia* at t_1._ AN: elevated branched-chain fatty acid.	It demonstrated that, upon weight gain, microbial richness increased, but perturbations in intestinal microbiota and SCAFs profiles in addition to several gastrointestinal symptoms did not recover.
			55(t_1_)		SCFAs and pH of feces (chromatography)			
			44 (t_2_)					
			55 normal weight HC					
Kleiman, 2017 [32]	Case series of longitudinal studies Grade: ***	DSM-5	*N* = 3 AN females	7 phyla and specific genera: *Faecalibacterium*, Ruminococcaceae, *Blautia*, Lachnospiraceae, *Clostridium*, *Streptococcus*, and *Bacteroides.*	Fecal sample	N/A	Significant changes in composition and diversity of the gut microbiota over time.	It found significant, patient-specific changes in the composition and diversity of gut microbiota during hospital based renourishment of 3 AN patients.
Mörkl, 2017 [33]	Cross-sectional observational Grade: **	ICD-10	*N* = 106 female 18 AN 20 athletes 26 normal weight 22 overweight 20 obese	Gut bacteria with special regards on Coriobacteriaceae.	Stool sample	HAM-D	Alpha diversity particularly lower in AN and obese. Alpha diversity correlated with BDI scores (i.e., depression correlated with a low number of observed species). AN: richer Coriobacteriaceae phylotype. AN: alpha diversity also correlated with dietary intake, SAT thickness, smoking, and serum lipids.	It sheds light on characteristics of the gut microbiome in different BMI and physical activity groups showing that a complex relationship between gut microbiota, body composition, diet, depression scores, and serum lipids.
					Anthropometric assessments	BDI-II		
					Ultrasound measurement of SAT thickness			
					Bioimpedance analysis			
					Laboratory parameters			
Borgo, 2017 [34]	Cross-sectional observational Grade: **	DSM-5	N = 30	Firmicutes, Bacteroidetes, Proteobacteria, Actinobacteria, and Verrucomicrobia.	Fecal sample	SCL-90	AN: significantly higher Enterobacteriaceae and *M. smithii.* AN: Butyrate inversely correlated with anxiety, propionate directly correlated with insulin levels, and relative abundance of *Roseburia inulinivorans.* BMI represented the best predictive value for gut dysbiosis showing a negative correlation with psychopathological scores (anxiety and depression).	It corroborates that gut dysbiosis could take part in neurobiology of AN, sustaining the persistence of alterations and relapses after renourishment and psychological therapy.
			15 AN 15 HC		Blood sample			
					Bioimpedance analysis	EDI-2		
					SCFAs	STAI-Y		
						BDI-II		
Speranza, 2018 [35]	Pilot study- observational Grade: **	DSM-5	N = 18	Not specified.	Fecal sample	N/A	AN-R: Butyrate and propionate significantly reduced. ANR: lower energy intake and REE.	AN had a reduced excretion of fecal SCFAs, likely as a mechanism to compensate for the lower energy and carbohydrate intake.
			10 AN-R		SCFAs			
			8 HC					
					Dietary record			
					REE (indirect calorimetry)			
de Clercq, 2019 [36]	Case report Grade: *	Not specified	*N* = 2	Firmicutes, Bacteroidetes, Verrucomicrobia, Euryarchaeota, Actinobacteria, Proteobacteria, Cyanobacteria, and Tenericutes.	Stool sample for FMT	N/A	Weight gain: 6.3 kg (from 45.8 to 52.1 kg) through 55% increase in body fat despite stable caloric intake. Increase in weighted phylogenetic diversity of gut microbial at 6 and 12 weeks, with marked increase of Verrucomicrobia. Significant increase in fecal acetate and butyrate levels after FMT, which might explain the change in metabolic rate.	The study showed that FMT induced weight gain in a patient with recurrent AN, suggesting that gut dysbiosis may be one of the causal factors in the etiology of persistent underweight in AN.
			26-years-old severe					
			AN female 1 unrelated healthy female donor		Changes in microbiota composition, metabolic parameters, and body composition assessed at baseline, 6, 12, and 36 weeks			
Hanachi, 2019 [37]	Case–control observational Grade: **	DSM-IV	*N* = 55	Not specified.	Fecal sample	N/A	AN: marked dysbiosis and lower alpha diversity. AN: higher *Klebsiella*, and *Salmonella.* AN: lower *Eubacterium* and *Roseburia.* Lower Peptostreptococcaceae in patients with FIDs. Undernutrition negatively correlated with Verrucomicrobiaceae and Ruminococcaceae, and positively correlated with Clostridiales order and Eubacteriaceae families.	Gut microbiota dysbiosis in malnourished patients with AN is correlated with the severity of FIDs and the severity of undernutrition.
			33 AN 22 HC		Francis score (for FIDs)			
					Blood sample			
Mörkl, 2019 [38]	Pilot study–observational Grade: **	ICD-10	*N* = 38	Not specified.	Fecal sample	N/A	AN: significantly lower alpha diversity. Similar Zonulin in AN and HC. No significant correlations between serum parameters and alpha diversity.	Decreased alpha diversity can have an additional negative impact on calorie intake in AN by changing in nutrient absorption.
			18 AN		Blood sample			
			20 normal weight HC		Zonulin as indicator of gut barrier function and inflammation parameters			
Prochazkova, 2019 [39]	Case report Grade: ***	Not specified	*N* = 2	Bacteroidetes (*Prevotella*), Firmicutes (*Roseburia*), *Ruminococcus, Blautia, Faecalibacterium prausnitzii, Clostridium, Anaerostipes, Eubacterium, Akkermansia muciniphila*, and *M. smithii.*	Stool sample for FMT	EDE-Q	Pre FMT: very low bacterial alpha diversity, lack of beneficial bacteria, and great abundance of fungal species. After FMT: both bacterial species richness and gut microbiome evenness increased. Fungal alpha diversity decreased. Gradual increase of total SCFAs levels. Serotonin: tended to decrease throughout the observation period.	FMT led to the improvement of gut barrier function. The need for an in-depth analysis of the donor’s stool and correct selection pre-FMT is evident.
			A 37-year-old AN female			BDI-II		
					PCR assay	BAI		
			1 donor (67-year-old first-degree female relative)		Metabolomic analyses pre- and post-FMT			
Monteleone, 2020 [40]	Longitudinal Grade: ***	DSM-5	*N* = 41 female	*Bacteroides*, Firmicutes, Actinobacteria, Coriobacteriales, Catabacteriaceae, *Clostridium*, and *Roseburia.*	Fecal sample	EDE-Q	AN: decreased intraindividual bacterial richness that disappeared after weight restoration. AN: increased Bacteroidetes-to-Firmicutes abundance ratio at t_1_.	Data showed a profound perturbation in the gut microbiota composition of AN patients. Moreover, gut bacteria in AN are connected with several fecal metabolites in a different way from HC and with divergent directions in the acute phase with respect to the weight-restored phases.
					PCR assay	BSI		
			21 AN (t_1_)		Fecal metabolomic (i.e., fat, coprosterol, fatty acid, SCFAs, sugars, and amino acids)			
			16 AN (t_2_) 20 HC					

Notes: GRADE: *very low; **low; ***moderate; ****high.

Abbreviations: α-MSH, α-melanocyte-stimulating hormone; AN, anorexia nervosa; AN-P, anorexia nervosa purging type; AN-R, anorexia nervosa restricting type; BAI, Beck Anxiety Inventory; BED, binge eating disorder; BDI-II, Beck Depression Inventory; BMI, body mass index; BN, bulimia nervosa; BSI, Brief Symptom Inventory; ClpB, caseinolytic peptidase B; EDE-Q, Eating Disorder Examination-Questionnaire; EDI-2, Eating Disorder Inventory-2; FIDs, functional intestinal disorders; FMT, faecal microbiota transplantation; HC, healthy control; HAM-D, Hamilton Rating Scale for Depression; IgM/IgG, immunoglobulins M/G; MADRS, Montgomery–Asberg Depression Rating Scale; MALDI, matrix-assisted laser desorption/ionization; PCR, polymerase chain reaction; REE, Resting Energy Expenditure; SAT, subcutaneous adipose tissue; SCFAs, short-chain fatty acids; SCL-90, Symptom Checklist-90; STAI-Y, state-trait anxiety inventory.

Although most of the studies used the *Diagnostic and Statistical Manual of Mental Disorders* (DSM-IV [14,25,26,29,37] and DSM-5 [32,34,35,40]) or the *International Classification of Diseases* (ICD-10) [33,38], some studies did not specify according to which criteria the diagnosis was made [27,28,30,31,36,39]. We included seven observational studies [14,25,26,30,33,34,37], three longitudinal studies [29,31,40], four case reports [27,28,36,39], one case series of three cases that was also longitudinal in its design [32], and two pilot studies [35,38]. The samples included mostly patients with a diagnosis of AN. Only one study evaluated BN and BED patients [30]. All studies included a control group matched for age and gender [14,31,33,34,40], with the exception of case studies [27,28,32] and studies on FMT cases [36,39]. A group of overweight and/or obese participants were collected in three studies [25,26,33]. And FMT was analyzed in two studies that presented case reports of female patients with chronic and severe AN [36,39].

The gut microbiota was examined from stool samples in all included studies. Other assessments comprised SCFAs [34,35], fecal pH [14,31], culture growth and mass spectrometry [27], anthropometric measures, ultrasound measurement of subcutaneous adipose tissue thickness [29,33], caseinolytic peptidase B (ClpB) protein concentrations [30], or fecal metabolomics [40]. Regarding psychiatric measures, the Eating Disorder Inventory (EDI-2), Beck Depression Inventory (BDI-II), Montgomery–Asberg Depression Rating Scale (MADRS), Hamilton Rating Scale for Depression, Eating Disorder Examination-Questionnaire, State-Trait Anxiety Inventory, Symptom Checklist-90, Brief Symptom Inventory, and Beck Anxiety Inventory scores were evaluated in relation to changes in microbiota composition [29,30,33,34,39,40].

**Figure 2.**
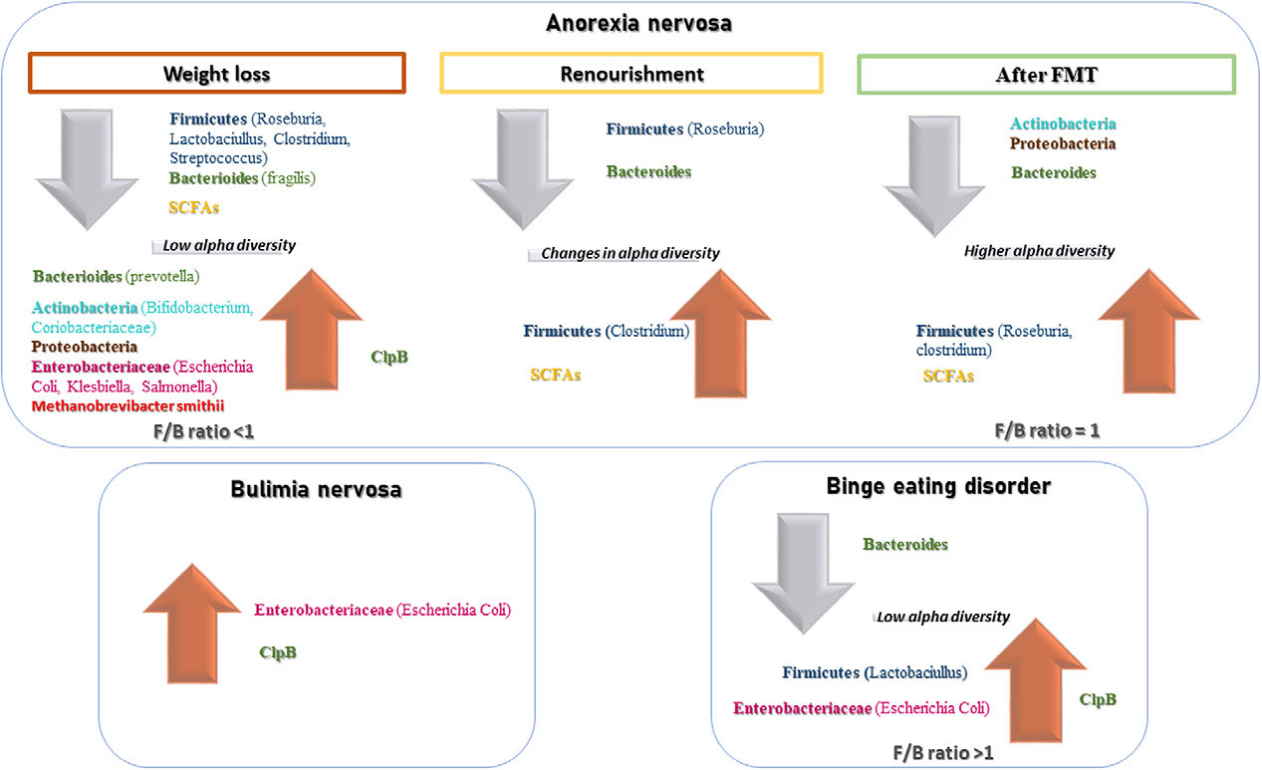
Main changes in gut microbiota composition in EDs.

In AN, significant changes in the quality, quantity, and composition of gut microbiota were found during weight modification. Alpha diversity was lower during the phase of weight loss [29,33,37–40], resulting in a reduction of Firmicutes [14] and SCFAs [14,34,35] and the increase of Bacteroides, Actinobacteria, Enterobacteriaceae, and *Methanobrevibacter smithii.* A re-established Firmicutes/*Bacteroides* (F/B) ratio and an increase of SCFAs levels were reported during renourishment and weight gain [29,31,32]. Data on BN and BED are limited but point in the direction of low alpha diversity and increase Firmicutes and Enterobacteriaceae. Interestingly, elevated ClpB concentration produced by *E. coli* suggested a role in stimulation and an autoimmune response, affecting the melanocortin system that regulates feeding behavior [30]. The FMT led to an increased number of bacterial producing SCFAs, alpha bacterial diversity, richness, and gut microbiome evenness increased in the AN patient [36,39]. For an overall view, see Figure 2.

## Discussion

Microbiota homeostasis is essential to promote a healthy gut, ensuring its structural [41], immune protective [42], and metabolic functions [43–45]. The imbalance between pathogenic and symbiotic or commensal species, so-called dysbiosis, seems to contribute to the development of EDs through several mechanisms [11–16] (see Supplementary Figure 1). This systematic review analyzed the contribution of dysbiosis to the pathophysiological development of EDs and the restored microbial balance as a possible treatment for EDs.

We found noteworthy evidence that quality and quantity of gut microbiota change in different way according to phase of AN disorder as well as indirect measures of microbiota diversity. SCFAs levels, lower during the restriction phase, increase during renourishment and weight regain. Unfortunately, only few studies had a longitudinal design, and results of cross-sectional studies should be read with attention, considering the absence of a control group. The role of microbiota in the etiology of BN and BED should be better deepened, conducting longitudinal trials. According to a transdiagnostic model of EDs, it would be interesting to identify possible markers of switch from AN to BN or BED, and vice versa, and to use them as therapeutic targets.

### Gut–brain axis

The two-way communication network between microbiota and the Central Nervous System [19,46] is finely controlled and regulated by neurotrophic substances (e.g., gamma-aminobutyric acid, melatonin, serotonin, catecholamines, acetylcholine, and histamine) synthesized by the intestinal microflora that “mimic” the endogenous molecules physiologically produced by our body [47]. Several authors have underlined the importance of the role of gut microbial balance for metabolism and appetite regulation [11,48–50]. Dysbiosis alters the hypothalamic–pituitary–adrenal axis [51], the hunger/satiety regulatory system [11,52,53], and mood [54]. Neuropeptide-like proteins produced by altered microbiota in EDs can simulate the endogenous appetite and satiety hormones [11,52] and cause a cross-reaction of immunoglobulin produced in stressful conditions (as in EDs) [55], such as autoantibodies against α-melanocyte-stimulating hormone (α-MSH). Studies have shown that high stress and psychopathological symptoms in patients with AN and BN are directly related to an increase in these anti-α-MSH antibodies [50,56,57] that act directly on the arcuate nucleus and on appetite regulation [58] (see Supplementary Figure 2).

### Anorexia nervosa

The possible role of the microbiota in the pathogenesis of AN has become a topic of recent interest [12,13], considering the close relation with gut microbiota composition according to the dietary intake in both the short term [59] and the long term [60]. Microbial diversity seems to change during diet restriction and also during weight regain.

#### Microbial diversity during diet restriction, renourishment, and weight regain

Significant changes in the composition of gut microbiota (in terms of phyla quality and quantity) on the basis of caloric intake were evaluated in patients with AN during weight loss [26,31,34,37], renourishment [32,40], and weight gain [26]. A significant decrease in Firmicutes levels [31,37], especially *Roseburia* [34], *Lactobacillus*, *Streptococcus* [14], and *Clostridium* [14,37], was found in patients with AN compared to healthy controls (HCs), independently of subtype (restrictive or binge/purge) or any compensatory behavior [14]. The majority of studies included in this review showed that alpha diversity in AN patients was lower [29,33,37–40] and suggested to interfere with nutrient absorption and calorie intake [38]. In humans, a lower F/B ratio was reported in underweight subjects compared to normal-weight subjects [61]. A diet with low carbohydrate or low lipid intake, as with that of AN patients, can favor an increase in Bacteroides [62], Actinobacteria, Proteobacteria, Enterobacteriaceae [31,34], *E. coli* [26,34], and *M. smithii* [25]. *M. smithii* (methane producers) seem to increase significantly in the intestinal epithelium of malnourished patients, providing a more efficient and increased supply of energy and calories [25,63,64]. Indeed, studies have demonstrated that acute malnutrition is characterized by gut dysbiosis and that the malnutrition phenotype can be transmitted via the intestinal microbiota in a gnotobiotic mouse model [65,66]. The imbalance in excess of commensal species in the patient’s intestine is a result of the restriction in food intake and may also account for the maintenance of the disorder. This microbial dysbiosis may interact with a nutrient-deficient diet affecting energy metabolism and causing the persistence of malnourishment. A reduction in microbial biodiversity also leads to an alteration of the immune system and to the assimilation and accrual of calories from food [13,67]. On the other hand, the systemic inflammation, as a result of the increased gut permeability, [68] and an altered neuronal activity are reported in various psychiatric disorders [15,69], and the same mechanism may be proposed for AN. More recently, 11 completely new bacterial species [27] and 4 new microeukaryotic communities never previously found in humans [28] were identified. Future studies should investigate new species and their contribution to gut microbiota stability.

Patients’ BMI and physical activity also showed a complex relationship with gut microbiota: patients with AN, similar to individuals during intense physical activity [70], have shown high levels of Coriobacteriaceae in their stool samples [33]. It has also been hypothesized that moderate and controlled exercise can provide an improvement in gut inflammation, decrease intestinal permeability, and positively regulate the composition of microbiota enhancing the number of beneficial microbial species [71].

Nutritional rehabilitation represents one of the essential focuses for EDs and has an impact on the composition of microbiota. The intake of macronutrients, such as fatty acids, proteins, and carbohydrates, especially fibers, can significantly affect the gut microbiota composition [17], facilitating the onset of dysbiosis [18] or restoring it. Weight regain with unbalanced diet, as high-lipid nutrition, in AN patients showed a reduction in Firmicutes and an elevation of *Bacteroides* and *Ruminococci* [29,31], an increase of mucin-degrading bacteria, and a reduction of butyrate-producing bacteria [72]. Interestingly, the microbiota changed qualitatively and quantitatively, and microbial richness increased after weight regain in patients with AN [29,31,32], but in some patients the trend of changes in alpha diversity was lower compared to HCs [40] and gastrointestinal symptoms did not recover at the end of 3 months treatment [31]. This may be due to a diet rich in fibers and suggests that energy derived from macronutrients is crucial for modest alpha diversity. Another explanation may be that microbial richness is related to colonic transit time [73]. It is well known that patients with AN often suffer from constipation and this could affect the measures for alpha diversity.

#### Short-chain fatty acids

SCFAs are fatty acids (butyrate, acetate, and propionate) produced by the gut microbiota during the nondigestible polysaccharides fermentation (fibers and resistant starch) [74]. The levels of SCFAs represent the indirect measurement of the microbial composition and are influenced by dysbiosis. SCFAs were lower in stool samples of AN patients [14,34,35], especially in the AN-Restricter subgroup, compared to the control group [14]. The reduction of SCFAs in patients with AN is a consequence of the low abundance of *Roseburia* [31,34]. A significant increase in both fecal acetate and butyrate levels during renourishment of AN patients [29] and changed microbiota composition, especially increased Firmicutes, after weight regain [31] were demonstrated. Bacterial species richness, gut microbiome evenness, and SCFA levels gradually increased in severe or chronic AN patients and also after FMT from a healthy donor. FMT resulted in an increase of specific genera and total SCFA levels, especially butyrate-producing *Roseburia*, 1 year after FMT, contributing to the improvement of gut barrier function [36,39]. A beneficial role in appetite regulation [75] as well as the involvement of SCFAs in energy homeostasis regulation has been suggested [76]. The importance of SCFAs on appetite and energy metabolism suggests SCFA modulation as new nutritional target to prevent or counteract EDs. Nevertheless, it is important to remind that the analysis of the fecal microbiota only indirectly reflects the upper intestinal flora and that the weight loss process involves consumption of metabolites instead of production [40]. Studies demonstrated also a significant enrichment difference in the gut microbiota composition according to the gut section [5]. Analysis of these microbial metabolites will surely improve the understanding of the etiology of EDs.

### Bulimia nervosa

If more data are available for AN, a dramatic lack of data is evident for BN. To date, only one study focused on microbiota changes in BN even if it is a life-threatening condition. Appetite regulation mechanisms seem modulated by changes in the microbiota even during BN [49,50,77,78]. ClpB was detectable in plasma of both HC and ED patients, but plasma levels were more elevated in the patients’ group. ClpB produced by *E. coli* is capable of “mimicking” α-MSH [79] and stimulating an autoimmune response [80]. IgG autoantibodies against α-MSH allow internalization of the IgG/α-MSH immunocomplex [30]. Furthermore, this mechanism has been highlighted in both AN and BN [81]. Therefore, a “hunger” rather than a “satiety” effect is due to the epitope switch of the IgG forming the immunocomplex in BN patients [82]. Binding occurs at the N-terminal in AN and at the C-terminal in BN, and in both cases is associated with anxiety and a high EDI-2 total score [77,82]. This cross-reactivity of α-MSH autoantibodies may also explain the possibility of shifting from AN to BN or BED [81,82], and vice versa, through modulation of the melanocortin system that regulates feeding behavior. Funding new evidence on the role of microbiota and its alteration in BN is the new challenging to address. Future research should investigate composition in patients with BN compared to HCs and other EDs and evaluate change in gut microbial species in prospective studies.

### Binge eating disorder

Studies regarding BED are still lacking. However, the relationship between microbiota and BED remains in the shadows, awaiting further research. A similar but opposite mechanism to that already described in AN has been hypothesized due to the cross-reactivity of IgG toward α-MSH in overweight [50,82] and obese patients [81]. Plasma concentrations of ClpB in patients with AN, BN, and BED were higher compared to HCs, without any significant differences according to diagnosis, suggesting a link between bacterial ClpB and EDs [30]. Serum concentrations of inflammatory cytokines and growth factors seem related to dysfunctional eating behaviors at the extremes of BMI, including BED [83]. As noted, BED is very often associated with numerous comorbidities, especially obesity [84], and patients with BED and obesity exhibited an unfavorable metabolic and inflammatory profile related to their characteristic eating behaviors [85]. It is evident that microbiota in obesity, similar to AN, differs in comparison to healthy, normal-weight subjects [26,33]. A diet rich in lipids is able to raise levels of Firmicutes and Proteobacteria and decrease levels of Bacteroides [86], thus leading to an increased F/B ratio [87]. A lower alpha diversity has been demonstrated in gut microbiota in both underweight and obese patients, with a significant correlation with BDI score indicating greater levels of depression [33]. In this light, the evidence of dysbiosis in gut microbiota with extreme BMI could justify its possible role in BED. In a transdiagnostic view of EDs [88], the hypothesis of neuroinflammation and gut dysbiosis in the etiology of EDs should be screened. Recently, a new a continuum model was presented, suggesting that changes in proinflammatory cytokines, serotonin levels, and microbiota cause shifts in EDs [89].

## Therapeutic approach: FMT

FMT is a new and promising therapy, already indicated in the treatment of diarrhea caused by *Clostridium difficile* [90–92], inflammatory bowel disease and ulcerative rectocolitis [92], autoimmune diseases, allergic syndromes [93], neurological syndromes such as Parkinson’s, multiple sclerosis, and fibromyalgia, as well as metabolic diseases (i.e., obesity, insulin resistance, and metabolic syndrome). The first FMT was performed in 2018 on a 26-year-old patient with severe restrictive AN after 2 years of standard therapies. Following FMT from a healthy donor, at a 36-week follow-up, the patient had gained 6.3 kg. The authors hypothesized that the patient’s microbiota remodeling could be due to the increased number of bacterial elements producing SCFAs, detected in the donor sample, in comparison with the basal levels recorded in the recipient [36]. More recently, another case report detailed the use of FMT on a 37-year-old female with severe chronic AN from a 67-year-old first-degree female relative donor [39]. Pre- and post-treatment analyses showed changes in gut microbiota composition: alpha bacterial diversity, richness, and gut microbiome evenness increased in the patient; and the fungal alpha diversity decreased, persisting for 1 year. Restoration of the F/B ratio can rehabilitate homeostasis or regulate the composition of the gut microbiota and correct abnormal responses of the mucosal immune system to chronic gut inflammation [94]. Despite bias, the results remain encouraging and indicate that, if replicated in a controlled trial, FMT may represent a new line of treatment in patients with AN; perhaps its use could be studied in other EDs. Although these results are promising, it should be taken into account that FMT is also associated with adverse events such as diarrhea, constipation, infections, and others not yet known.

## Microbiota and outcome

Gut–brain interplay is fundamental, and numerous intestinal microflora have an important role through the synthesis of neurotransmitters [47]. Unfortunately, there is little evidence supporting the associations between the intestinal microbiota and depression or anxiety disorders in humans [95,96]. In this systematic review, few studies have cleared up the relationship between dysbiosis and psychopathology in AN [29,30,33,34,39,40], with only one study in BN and in BED [30].

Depression, anxiety symptoms, and ED psychopathology seem to be related to a change in diversity of gut microbiota, especially bacterial species producing butyrate [12,33,34,97]. In particular, a lower bacterial diversity is associated with more severe depression and anxiety [29,30,33,34]. Longitudinal studies on AN cohorts suggested that weight gain during renourishment leads to the improvement of psychological symptoms with specific changes in microbial composition that might participate in AN pathophysiology [29]. Serotonin secretion regulation and the role of gut microbiota during its synthesis in the intestine are well documented [98,99]. It could be speculated that variableness in gut microbiota in EDs may affect the expression of the tryptophan hydroxylase protein or serotonin transporters and result in consequential abnormalities in serotonergic activity and psychopathological symptoms [97]. The altered homeostasis in gut microbiota seems related to the altered secretion of serotonin [100] and the cross-reactive mechanisms through the productions of autoantibodies against neuropeptides [80]. As above mentioned, the ClpB concentration in BN and BED stimulates the production of autoantibodies against α-MSH. The different binding of ClpB has been associated with psychological traits in BN and BED patients, especially with more anxiety, high MADRS, and EDI-2 total score [77,82], supporting a link between bacterial ClpB and ED pathophysiology [30]. In a recent case report, serotonin levels tended to decrease throughout after FMT [39], but mood and eating pattern of purgative AN remained unmodified, despite significant improvement in the microbiota post-FMT [39] probably due to the long duration of illness or a short follow-up. On the other hand, patients with AN were treated with antidepressants, which are known to induce microbiota alterations due to their antimicrobial activity [101] and could be responsible for contrasting results.

## Future directions

New therapeutic options in the clinical management of EDs are currently being investigated as direct and/or adjunctive therapies [102]. Among them, the so-called psychobiotics have been studied more extensively. These are probiotic live organisms that, when ingested in adequate amounts, produce a health benefit in patients suffering from psychiatric illness [103]. *Lactobacilli*, *Bifidobacteria*, *Enterococci*, and yeasts are used mostly in the formulation of probiotics [104] and are important for the production of SCFAs, the biosynthesis of vitamins B and K, the production of neuroactive substances such as gamma-aminobutyric acid and serotonin, the activation of the immune system and regulation of cytokine and immunoglobulin release, the reinforcement of intestinal barrier function through tight junctions, and the increase of mucin levels [103,105]. Studies on mice [106,107] were replicated in human volunteers [108] and demonstrated an increase in neuropsychiatric disorders after inducing dysbiosis that subsided after oral administration of probiotics. These results provided evidence of antidepressant and anxiolytic effects of probiotics probably due to a decrease in the levels of proinflammatory plasma cytokines [109].

*Enterococcus* and *Lactobacillus* seem to regulate the Enterobacteriaceae responsible for the production of autoantibodies against α-MSH [80]. Probiotics such as *Roseburia*, a butyrate producer [16,31], are among the proposals for future therapeutic protocols in the management of AN.

Other studies have confirmed how these supplements have been able to rehabilitate malnourished and hungry rodents, confirming the usefulness of this supplement in the treatment of malnutrition [110–112].

## Strengths and limitations

This represent a comprehensive systematic review on the role of microbiota in the pathogenesis and treatment in individuals with EDs. It is also the first to include studies on AN, BN, and BED in an attempt to explain the possible role of dysbiosis in the pathogenesis of these EDs. Nevertheless, some limitations should be noted. First, the heterogeneity among the included studies may be a confounding variable in interpreting results. Few studies have a longitudinal design, and the majority of studies are cross-sectional observational or case report studies that preclude conclusions about causality. We found only two studies evaluating FMT in AN to illustrate the therapeutic potential of this innovative treatment. Second, the study samples were mainly made up of female participants, which limits generalizability to males, and the variable sample size reduces the power to detect differences between patients and controls over the course of renourishment. Third, interpretation of the results of the included studies is hindered by limitations inherent in the confounding variables affecting dysbiosis: self-reported information on dietary intake, impact of starvation, short timeline of longitudinal studies, antidepressant treatment, and indirect analysis of microbiota through the fecal sample. However, the strongest limitation concerns the composition of a “normal” microbiota, which changes according to geographical area of origin, eating habits, food choice, gender, age, and type of birth delivery. Moreover, current scientific research focused mainly on bacteria and less on other microbial species. Randomized, placebo-controlled studies designed to unravel the gut–microbiota cross-talk mechanisms are therefore needed.

## Conclusions

Understanding the composition and functions of microbiota and dysfunctional mechanisms could be important in preserving and improving its balance. However, this is only the tip of an iceberg of complex interactions between the microbiota and the host. A healthy gut microbiota profile improves health and prevents several disorders. The importance of the diet in regulating the physiological balance between Firmicutes and Bacteroides is well known, showing how diets rich in fibers and biotic supplements can restore the normal commensal flora even in EDs. Sufficiently strong and solid scientific evidence has not yet been produced, but the activity of SCFA-producing bacteria, especially butyrate, is known to be fundamental in preserving the epithelial integrity of the gut. The depletion of these bacteria and the consequent increase in the number of mucin-degrading bacteria promote intestinal inflammation, permeability of the enteric epithelium, and therefore the passage of lipopolysaccharide and ClpB protein into the circulation. These can trigger immune reactions in the hunger/satiety regulation center by losing the ability to control food intake. The reduction of butyrate-producing bacteria and the increase of pathobiont bacteria, such as *Clostridium*, are related to cross-reactive mechanisms involving the HPA axis in patients with EDs, anxiety, and depression.

This systematic review highlights the extreme and delicate communication network between gut, endocrine system, and brain. A better and more in-depth characterization of gut bacterial species in the near future may provide useful indications to improve not only the therapeutic but also the preventive approach in EDs. Future research may be able to distinguish between changes of intestinal microbiota that reflect weight gain versus recovery from EDs and identify microbial biomarkers of renourishment versus recovery from psychopathology used as therapeutic targets. In this light, it may be possible to identify the patients who will benefit most from these new therapies and those who may not have any benefit at all. Just think of the latest proposed procedure, the FMT, where a desired phenotype could induce other disorders. A new promising therapeutic strategy may be the administration of probiotics or prebiotics for restoring the gut microbiota in patients with AN, BN, and BED. It is also increasingly evident that the gut microbiota represents a real “organ” with specific and fundamental functions for the protection and prevention of many disorders, as well as playing a possible role in their pathogenesis.

Further research may investigate in-depth microbiota changes with particular regard for BN and BED comparing to HCs. Future studies should also disentangle if there are differences in gut microbiota composition between obese patients after weight loss and AN and finally elucidate the possible role of new therapeutic strategies.

## Supplementary material

For supplementary material accompanying this paper visit https://doi.org/10.1192/j.eurpsy.2020.109. click here to view supplementary material
