# Supplementary figures and images for: A systematic review on the role of microbiota in the pathogenesis and treatment of eating disorders
Source: Eur Psychiatry. 2020 Dec 16;64(1):e2. doi: 10.1192/j.eurpsy.2020.109 (PMC8057489; doi:10.1192/j.eurpsy.2020.109)

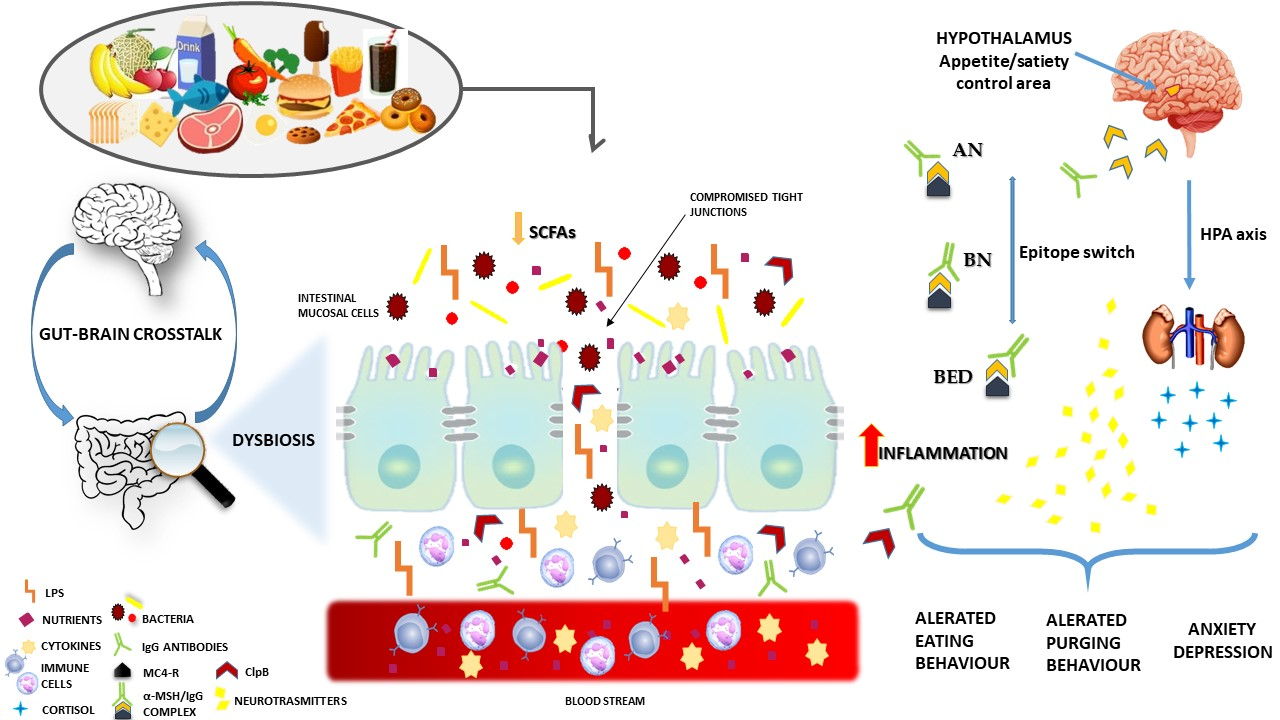

Supplement: Supplementary file 1 [file S0924933820001091sup.zip › S0924933820001091sup002.tiff]

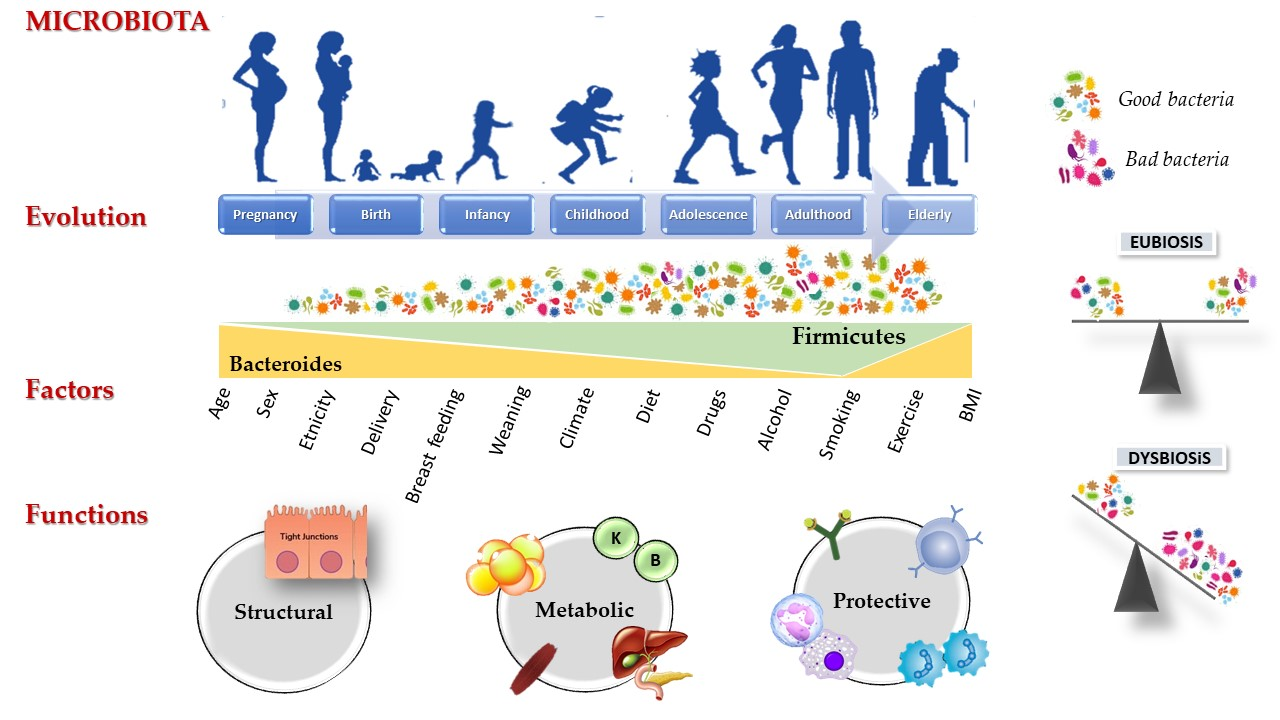

Supplement: Supplementary file 1 [file S0924933820001091sup.zip › S0924933820001091sup001.tiff]
